# Factors associated with motorcycle-related road traffic crashes in Africa, a Scoping review from 2016 to 2022

**DOI:** 10.1186/s12889-022-13075-2

**Published:** 2022-04-05

**Authors:** Kennedy Diema Konlan, Linda Hayford

**Affiliations:** 1 Department of Public Health Nursing, School of Nursing and Midwifery, University of Health and Allied Sciences, Ho, Volta region Ghana; 2 College of Nursing, Yonsei University, 50-1, Yonsei-ro, Seodaemun-gu, Seoul, 03722 South Korea; 3 Department of Medicine, St Anthony Hospital, Dzordze, Volta Region Ghana

**Keywords:** Road, Traffic, Accident, Crash, Injury, Motorcycle, Cycling, Commercial, Regulations

## Abstract

**Background:**

The toll associated with road traffic crashes (RTC) is high, and the burden of injury is disproportionately borne by pedestrians and motor riders, particularly in developing countries. This study synthesized the factors associated with motorcycle-related RTC in Africa.

**Methods:**

The PICO framework and the PRISMA guidelines for conducting reviews were incorporated in searching, screening, and reporting the findings. Advanced search in five electronic databases (Google Scholar, PubMed Central, Scopus, CINAHL, and Embase) yielded 2552 titles and 22 from manual search, filtered for 2016 to 2022 (to generate 1699) and then further for primary studies (854). Through the title, abstract and full-text screening, 22 were appropriate for this review. Data extraction was done by the two researchers independently, and the results were compared. Convergent synthesis was adopted to integrate results, transformed into a narrative, and analyzed using thematic synthesis.

**Results:**

The four main themes identified were the rider-related, non-rider-related factors, prevalence and severity of injuries from RTC, and the measures to reduce RTC. The behavioral factors associated with RTC were alcohol use, smoking, use of illicit drugs, tiredness of rider, poor knowledge on traffic regulations, more than one pillow rider, lack of rider license, non-observance of traffic regulations, and non-use of personal protective equipment. Road traffic crashes were common among younger age and male gender. Other factors identified included poor road network, unplanned stoppage by police, unlawful vehicular packing, increased urbanization, and slippery floors.

**Conclusion:**

There is the need to institute multi-sectoral measures that target riders’ behavior change. Coordinated efforts should target governments, enforcement authorities, and regulatory bodies to enforce enactment that ensures safe use of roads.

## Background

Motorcycling plays various roles in the life of people from all categories of life. This role played by motorcycles is for pleasure and transportation [1, 2]. Riding is a viable option of transport, with over 770 million motorcycles estimated on the roads [2–4]. In developing countries, motorcycles are used in serviceable responsibilities related to mobility, transport, sport, and economic activities [1]. Increasingly, in African peri-urban and urban centers, the motorcycle is becoming the de facto means of transport [1, 2, 5, 6]. The increased preference of the motorcycle as means of transport is because it is compact, agile, fuel-efficient, and easy to maneuver in congested areas [1, 3]. The toll associated with road traffic crashes (RTC) is high, and the burden of injury is disproportionately borne by pedestrians and riders, particularly in developing countries [2, 7]. Motor riders represented more than 380,000 annual deaths worldwide and accounted for over 28% of the global fatalities of crashes in 2016 [3]. Road traffic injury (RTI) death rates are highest in Africa and other developing countries [3, 8, 9]. The occurrence of road traffic accidents (RTA) and severe crash injuries involving commercial riders has risen significantly in recent times [4]. Some common injuries associated with RTC are spinal and head injuries that leave in its trail a long time disability [10, 11].

Researchers attributed several behavioral and societal factors such as rider’s age, gender, circadian rhythms, riding experience, type of road, and characteristics of the motorcycle to increase the risk of RTAs among riders [1, 3, 4, 12]. Motor riders are exposed to excessive physical demands during riding that may have an impact on fatigue level [3, 4]. Incidence of fatigue is substantially higher among riders than drivers of other modes of transport [1]. Another factor associated with the increasing rate of RTC is the use of unauthorized or illicit drugs among riders [4, 13]. In Africa, commercial motorcycle riders are usually poorly educated, have limited training on riding, and maybe engage in illegal drug use [4, 13, 14].

Research on RTI has not received adequate attention from the scientific community in low- and middle-income countries [2, 5]. There are still gaps in available data for formulation of policy and reducing associate risk to RTC. Various studies conducted in Africa are localized to only specific geographic regions and only assessed factors related to road safety or injuries among riders, pedestrians, first aid givers, and commercial drivers [14–17]. These studies also, on a few occasions, assessed the factors associated with the damages resulting from RTC or type of treatment received by victims as well as the means of transport [14–17]. There is, therefore, the need to have one document that synthesizes the factors that are associated with RTC, especially in Africa. This is because various reviews conducted in Africa are sporadic and mainly country-based or assessed the trend of injuries, road, and infrastructure, impacts of RTCs, access to hospital and emergency centers, and nature of RTIs [4, 17]. This study identified and described the factors associated with motorcycle-related RTC in Africa.

## Methods

### Literature search

A scoping review incorporating the Population, Intervention, Control, and Outcomes (PICO) framework of the literature search was used. The Preferred Reporting Items for Systematic Reviews and Meta-Analyses (PRISMA) guidelines were incorporated in searching, screening, and reporting scientific results [18–20]. There was an advanced and comprehensive search of five databases through the main library website of Yonsei University. These databases included Google Scholar, PubMed Central, Scopus, Cumulative Index to Nursing and Allied Health Literature (CINAHL), and Embase using Medical Subject Headings (*MESH terms)* derived from the keywords. Medical Subject Headings is the National Library of Medicine controlled vocabulary thesaurus used for indexing articles for PubMed. Keywords that were the embodiment of the study title and their derivatives served as the guide for the search. The keywords and related synonyms were searched using the appropriate Boolean operators. The associated keywords and synonyms were (“factors associated” ‘OR’ causes ‘OR’ determinants) ‘AND’ (“Commercial motorcycle” ‘OR’ motorcycling ‘OR’ motorbike) ‘AND’ (“Road traffic accident” ‘OR’ “road accident” ‘OR’ “road crashes”). Following the search, 2552 titles were identified through the electronic search and 22 from manual search (of reference list of identified studies), filtered for the last 6 years (2016 to 2022) to yield 1699, and then further for only primary studies (854 titles).

**Fig. 1:**
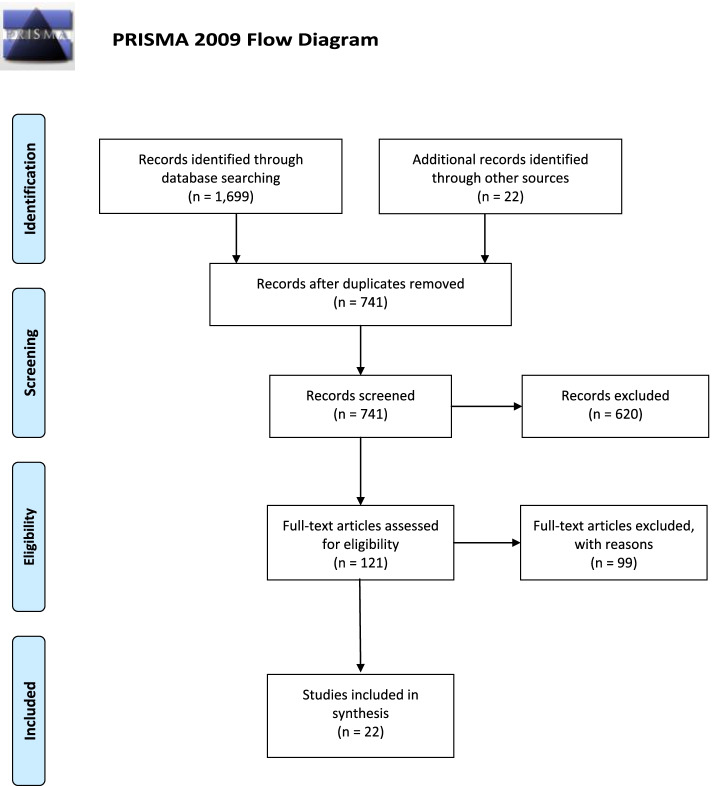
PRISMA flowchart for identification and selection of articles

All the 854 identified titles were transported to endnote version 9X, and duplicates were identified and removed to produce 741 titles. These identified titles (741) were screened for appropriateness by the two researchers independently, and 121 abstracts were selected to be related to or identified the factors that are associated with RTC. During the title and abstract screening, when the researchers were not sure of including a particular study, it was added for full-text screening. In instances the two researchers had varied opinions of the inclusion of a specific article for the next stage, a third person was always consulted, and majority decision prevailed. Eventually, 22 articles were settled on as appropriate for this study after full text screening, as shown in Fig. 1. The reasons for the inclusion of these studies were because they were primary studies involving motor riders (both for private and commercial use), the central theme discussed the factors that are associated with RTC, were conducted in Africa, and published within the stipulated time.

### Inclusion and exclusion criteria

The studies that were included in this review were conducted in Africa; primary studies, identified factors that are associated with motorcycle crashes, were English-based articles and published between 2016 to 2022. Studies that determined the nature of the injury, type of treatment, time to receive treatment upon an RTC were excluded. Also, non-English, and non-primary studies were also excluded.

### Data extraction and analysis

Using a matrix developed by the researchers, data extraction was done by the two researchers independently, and the results were compared. Discrepancies were discussed until there was a consensus. In instances where an agreement was not reached after several repeated discussions, a third person was consulted—the comparison of the extracted data between the two extractors allowed for streamlining of any ambiguity. Upon the involvement of the “arbiter” all discrepancies were resolved through consensus. The main parameters that were extracted were country, purpose, population and sample, design, analysis, key findings, nature of injuries, conditions of RTI, and interventions to reduce RTC. The convergent synthesis design was adopted to integrate results from studies and transform them into narrative findings [21]. A thematic approach was used to synthesize key findings emerging from the literature in relation to the factors that are associated with RTC among riders [22]. The researchers used line-by-line coding of various studies independently to enhance the identification of free codes. The codes identified were then collated into subthemes and finally into main themes [22, 23]. The themes that emerged from the analysis included rider-related factors, non-rider-related factors that influence RTCs, prevalence and severity of injury from RTCs, and the measures to reduce RTCs among riders.

## Results

The study populations were registered commercial motor riders [2, 24–28], motor riders [14, 15, 17, 29], persons involved in motorcycle crashes [30], community members [16], and patients attending the emergency department of the hospital due to RTI [31, 32]. The sampling methods employed were convenient sampling [14, 32], purposive, and convenience sampling [17]. The probability sampling methods included simple random [15, 25, 31], cluster, and systematic sampling [2, 16, 24]. Others used police-reported data on RTCs that resulted in injuries [30]. The studies were conducted in Ghana [2, 16], Kenya [28, 33], Cameroon [14, 25], Tanzania [30, 34], Uganda [15, 17, 24, 32, 35] and Ethiopia [31]. All studies were quantitative studies except one that was a mixed-method approach [15]. The specific study designs adopted included matched case-control [32, 36], descriptive cross-sectional [2, 16, 17, 24, 25, 31, 37], retrospective analysis [30, 38], and correlational designs [15]. Data analysis methods adopted included descriptive analysis [2, 28, 35], chi-square [2, 24], bivariate and multivariate regression [15, 16, 24, 32], multivariate analysis and log-normal model [16, 24, 27, 29, 31], and conditional logistic regression [32, 34, 36]. Also, there was an independent t-test and Chi-square [2, 15, 17], Fisher’s exact test, and Hosmer-lemeshow goodness-of-fit statistic [25]. The studies were funded by educational and research institutions that included the world bank global road safety facility grant [32], Department of Geography, University of Western Ontario, Canada [16], University of California San Diego international institute, global health institute [25], Fogarty international centre [30], Institute for management and professional training [14, 17], and Addis Ababa university [31].

## Main themes

It was noted that four main themes were identified from the synthesis of data. The four main themes identified were the rider-related, non-rider-related factors, prevalence, and severity of injuries from RTC, and the measures to reduce RTC. The themes were formulated through the synthesis of the critical findings in Table 1. These themes and subthemes are shown below.

**TABLE 1:** SUMMARY OF KEY FINDINGS

AUTHOR	PURPOSE	SAMPLE	KEY FINDINGS
Tumwesigye et al., 2016 [32]	Established the factors associated with cycle injuries	289 cyclists per arm.	Independent factors associated with RTI included younger age, current alcohol intake, lower bike engine capacity, riding experience less than three years, riding over long periods, old motorcycle, sharing motorcycle, low-level knowledge of traffic rules (TR), and police stop for checks.
Konlan et al., 2020 [2]	Determined the prevalence and pattern of RTC among commercial cyclists	114 commercial cyclists	Prevalence of RTC was 64 and 74% RTC in the past one year RTC was attributed to excessive speeding, alcohol use, reckless riding, bad roads, collision with another cycle, slippery surfaces, non-observation of traffic regulations, wrongful overtaking.
Vaca et al., 2020 [34]	Provided an overview of Ugandan traffic safety trends in the past decade, focus on *boda bodas* (cyclist)		Police report: Male were frequently involved in RTC (73.95%) RTC were associated with younger adults (25-34 years), careless and reckless driving RTC is more localised in the capital. Hospital data: RTC victims were 59% (July 2015) and increased to 72% in 2018. RTI accounted for 41% of trauma, and head traumas are 54% (2015) and 62% (2018)
Konkor et al., 2019 [16]	Examined risky behaviours and timing to first collision among cyclists	818 household representatives	Experienced RTC (50%) The average timing to the first RTC was 5.3 years. Knowledge on safety of helmet use and the speed limit was low Alcohol consumption and knowing someone who died of RTC were higher among those with a previous history.
Wankie et al., 2021 [25]	Estimated the prevalence of RTC and contributing factors	557 commercial cyclists	Mean age of 28.7, currently smoking (12.6%), alcohol use (68%) involved in RTC RTC (77.4%), and average incidence was 3.3 crashes. Higher odds of RTC among riders with ≥3-5 years riding experience, carrying two or more passengers, alcohol use, poor roads, and speed above 45 km/hr.
Reardon et al., 2017 [30]	Described the epidemiology and geographic distribution of RTCs in Moshi, Tanzania.	300 RTIs - Police data from Feb 2013 to Jan 2014	Most injuries occurred at 4 intersections on 2 main corridors. Car crashes (48%) and motorcycle collisions (35%) mainly involve males. Cyclists (43%) wore helmets. RTC were grievous (12%), and the average victim age was 33 years. Occurred during daylight (67%) as 24% had alcohol test and 14% were positive
Ndagire et al., 2019 [24]	Determined compliance based on a combination of 4 safety measures and associated factors	340 motorcyclists	Mean age of 29.5 RTC victims and riding experience of 1 to 20 yrs., Cyclists had riding permits (47.1%) and had class A permit (28.8%), retro vest (39.1%), helmet (89.1%), and carried only one pillion rider (86.1%). Only 3 riders complied with all 4 safety measures
Muni et al., 2019 [17]	The risk of self-reported RTC is lower in safe Boda than in regular drivers	342 cyclists - 171 each arm	85 crashes- 31 in safe Boda and 54 in regular riders for 6 month follow up period Safe Boda drivers were 39% less likely to be involved in RTC than regular riders after adjusting for age, possession of a license, and education. Attended a road safety training (86.4%)
Havugimana et al., 2020 [15]	Influence of cyclists’ practices on road safety	384 riders and four key informants	Level of compliance with safety practices, valid license, and helmet use (27.2%). Personal factors - age, receipt of training, and attitude towards road safety practices, influenced compliance independently.
Abia & Tache, 2017 [14]	Knowledge of cyclist on road safety, safe riding practices, and the usefulness of PPE	300 riders from selected parking points	Had formal motorcycle riding training (20%) and basic PPE knowledge (95%). Never wore any PPE during motorcycle riding (65%). Riders had little or no knowledge of road safety, ethics, or the importance of PPE (80%). Riders were aware of road code and traffic signs (75%), though 40% did not respect it.
Baru et al., 2019 [31]	Factors affecting injury severity levels of RTC victims referred to selected public hospitals in Addis Ababa	363 RTC victims	Severe injury among RTA victims (36.4%). Victims extricated at the scene by health care professionals, police, and street traffic police control were significantly associated with less severe injuries. Cyclist or Pillion without a helmet used alcohol, had multiple injuries, collision in cross-section, unrestrained occupant, in the back of a truck was associated with severe injury
Agyemang et al., 2021 [29]	Investigated and compared factors associated with motorcycle crash injury outcomes in rural and urban areas	Five years of motorcycle crash records (2014 to 2018)	Rural area crashes occurred under dark and unlit roadways, and urban areas recorded more intersection-related crashes. Pedestrian collisions occurred in urban areas and head-on collisions in rural areas. Collisions with a pedestrian, run-off-road, and collisions under dark and unlit roadway conditions resulted in fatal injury.
Boniface et al. 2016 [36]	Determine the pattern, associated factors, and management of road traffic injury patients	4675 road traffic injury patients	RTC (70.2%) victims were between 18 and 45 years. Motorcycles were the leading cause of road traffic crashes (53.4%). Factors associated with mortality were; using police vehicles to hospital (P = 0.000), receiving medical attention within 2 to 10 h after injury (*P* = 0.000), 18–45 years age group (P = 0.019), not using helmet (P = 0.007), severe injuries (P = 0.000) and sustaining multiple injury (P = 0.000).
Dapilah et al., 2017 [39]	Examined how motorcyclist characteristics influence road traffic behavior and its implications	A randomized sample of 220 motorcyclists	Age, occupation, and motorcycle ownership were significantly associated with wearing a helmet. Age and alcohol use was found to have a significant relationship. The number of road traffic accidents and deaths were related to road traffic behavior of motorcyclist
Kiwango et al., 2021 [35]	Determined the association between alcohol consumption, marijuana use, and RTIs among commercial motorcycle riders	Cases (164) attending, Controls (400) not attended hospital	Risky drinking was associated with close to six times the odds of RTIs (OR = 5.98, 95% CI: 3.25–11.0) and remained significant after adjusting for sociodemographic, driving, and work-related factors (OR = 2.41, 95% CI: 1.01–5.76). The crude odds ratios of RTIs were significantly higher among users of marijuana (OR = 2.33, 95% CI: 1.38–3.95).
Ndwiga et al., 2019 [38]	Determined factors associated with road traffic accidents involving motorcyclists	180 commercial motorcyclists	Road traffic accidents (38%) in the past one year and at least once (69.1%) Motor riders attributed the occurrence of the accident to poor visibility (26.5%), overspeeding (23.5%), careless motorists (13.2%), and potholes (8.8%).
Ngari et al., 2019 [27]	Determined the incidence of commercial motorcycle accidents (MCAs)	202 commercial motorcycle riders	Riders in singlehood marital status were almost twice as likely to experience an MCA compared to those married [Adjusted HR (AHR) =1.8 (CI: 1.1, 3.4), *p* = 0.046]. Khat (*Catha edulis*) users were 2-fold likely to experience an MCA relative to non-Khat (*Catha edulis*) users [AHR = 2.1 (CI: 1.1, 4.2; *p* = 0.021].
Sahr et al., 2020 [26]	Examined the factors associated with the occurrence of road traffic accidents among motorcycle riders	Motorcycle riders (61) and passengers (59).	Accidents mainly occur due to riders riding a motorcycle without formal training Motorcyclists who were without driver’s license had lack of adequate professional riding knowledge, Other factors were mechanical defects, bad roads, over speeding, over-load, traffic officers, police harassment of riders
Salum et al., 2019 [33]	Identified the factors influencing the severity of motorcycle crashes.	784 motorcycles crashes that occurred from 2013 to 2016	Factors that increase the probability of fatalities were speeding, driving under the influence, head-on impact, presence of horizontal curves, reckless riding, off-peak hours, violations, and riding without a helmet. Crashes occurring on weekdays, during peak hours, at intersections, involving a rear-end impact, in daylight, on-street roads, and under clear weather conditions decrease the probability of a fatality.
Singoro et al., 2016 [33]	Determined the causes and trends of motorcycle accidents	400 people from households	Human error is the leading cause of motorcycle accidents. Structured and comprehensive training of riders on traffic codes and regulations will most likely reduce accidents and associated economic losses.
Vissoci et al., 2020 [28]	describe the safety behaviors of commercial motorcyclists	609 commercial motorcyclists	Motorcycle drivers (38.7%) experienced a crash during their lifetime, of which more than half (n¼134, 56.8%) suffered injuries. Motorcyclists (100%) reported always wearing a helmet, a chin strap (99%), and having a passenger helmet (98.8%).
Adeleye et al., 2019 [37]	Review the clinical epidemiology characteristics of motorcyclist	833 of all roads related injuries	Victims had a mean age of 33.1 years and consisted of males (83.1%), low socioeconomic status (> 90%), aged between 20 and 40 years old (56%). MCCs involved only riders (32.1%), and 69% were motorcycle crashes.

### Rider related factors associated with RTCs

Three sub-themes were identified and included riders’ behavior, knowledge of on-road use and regulations, and compliance with traffic regulations.

#### Rider behavioral related factors

The factors that were associated with RTC were the use of alcohol and other drugs by riders [2, 16, 25, 30, 32, 37, 39]. It was noted that 12.6% of riders currently smoke, and 68% typically use alcohol [25]. Alcohol use [39] was common among road users as 24% had alcohol tests, and 14% were positive [30]. Risky drinking was associated with close to six times the odds of RTIs (OR = 5.98, 95% CI: 3.25–11.0) and remained significant after adjusting for sociodemographic, driving, and work-related factors (OR = 2.41, 95% CI: 1.01–5.76) [36]. Human error was one of the leading causes of road traffic crashes among motor riders [28, 33]. Other behavioral factors included riding experiences of less than 3 years [25, 32], more extended periods of riding [2, 32], riding till late [32], sharing motorcycles between riders [32], excessive speeding [2, 39, 40], careless, and reckless riding [2, 29, 35, 40]. There were higher odds of crash among riders with more than 3-5 years of riding experience and typically with two or more passengers [25]. Age and gender of the rider influenced the risk of having a crash as young people (less than 30 years) had increased odds of an RTC [15, 25, 32, 35]. The mean age of RTC was 28.7% [25] and mainly involved male [29, 30, 35]. RTC (70.2%) victims were between 18 and 45 years and Motorcycles were the leading cause of road traffic crashes (53.4%) [37]. Riders who were unmarried had a higher probability of a road traffic crash [27]. Crashes occurring on weekdays, during peak hours, at intersections, involving a rear-end impact, in daylight, on-street roads, and under clear weather conditions decrease the probability of a fatality [34].

#### Riders’ knowledge of road traffic regulations

Knowledge levels were generally seen to below, especially in using personal protective equipment (PPE) by riders. Knowledge on the safety of helmet use and speed limits was low for those who experience RTC [16, 25]. Inadequate knowledge of traffic regulations increased the chance of an RTC among riders [2, 26, 29, 32, 40]. Only 20% of riders had formal motorcycle riding training, and 95% had basic knowledge on the use of PPE [14]. Also, 80% of riders had little or no knowledge of road safety, ethics, or the importance of PPE [14]. Persons who received training on road traffic regulations had a mean age of 32.8 years among the 86.4% who attended a road safety training [17]. Receipt of training and attitude towards road safety practices independently influenced compliance to road traffic regulations [15].

#### Compliance of riders to RT regulations

Most riders did not have a valid rider license [15, 24, 26] as the level of compliance with safety practices, including helmet use, was 27.2% [15]. Reported compliance to helmet use and ownership was 7.6 and 89.1%, respectively [24]. In other areas, the use of the helmet by rider was reported [28] at 43% [30]. The importance of license was manifest as safe riders (‘boda boda’) were 39% less likely to be involved in RTC than regular riders after adjusting for age, possession of a license, and education [17]. The use of helmets in the last trip was 69.4, and 86.1% carried only one pillion [24, 28, 40]. Also, 75% of riders were aware of road codes and traffic signs, though 40% did not comply [14]. It was also noted that 47.1% of riders had to ride permits [24]. Class A permit is owned by 28.8%, while 39.1% allegedly owned the retro vest, with a few using it on the last trip [24]. Factors that increase the probability of fatalities were speeding, driving under influence, head-on impact, presence of horizontal curves, reckless riding, off-peak hours, violations, and riding without a helmet [34].

### Non-rider-related factors that influence RTC

This theme identified the non-direct human factors that were related to the occurrence of an RTC among riders. In this theme, two sub-themes were identified and included motor and police-related factors and road-related factors.

#### Motor and police related factors

The factors related explicitly to the motorbike included lower engine capacity [32], not changing a motorcycle [32], and having too many motorcycles on the road [35]. The policy-related elements mainly concerned that the police stopped riders at a place that was not designated [2, 26, 32].

#### Road-related factors

Several other factors were identified to be road-related factors that influence the occurrence of RTC among riders. These factors include poor roads [2, 25, 39], parked vehicles at an unapproved point [2, 25], slippery surfaces [2], poor visibility [26, 39] and overspeeding (above 45 km/hour) [25, 34, 37, 39]. Other road-related factors were concerned with busy intersections having a high chance of RTC [26, 32, 34], riding on the main road [30], and RTC usually occurring in predictable high traffic locations [30].

### Prevalence and severity of injuries from RCTs

This theme described two sub-themes that included the prevalence of crashes and injuries related to RTCs and factors related to injuries resulting from RTCs.

#### Prevalence of crashes and injuries related to RTCs

The prevalence of RTCs was identified to be more common in urban settings in Uganda [35] and Tanzania [37]. In Ghana, the prevalence was 64%, with 74% involved in crashes in the past year [2], and about half have a history of RTC. The average timing to an RTC from the point of first riding was 5.3 years [16]. Also, knowing someone who died or was involved in RTC was higher among motorbike riders [2, 16]. It was also reported that car and motorcycle collisions were 35% [30] and 77.4%, with an average of 3.3 crashes and 21.5% involvement within 12 months [25]. The time of day of occurrence of RTC showed that 67% occurred during daylight hours [30]. Victim with multiple injuries, collision in cross-section, vehicle occupant traveling unrestrained on a back of a truck were related to severe injury [31, 38].

#### Factors related to injuries resulting from RTCs

Victims extricated at the collision scene by health care professionals, police, and street traffic police were significantly associated with less severe injuries [31]. Initial immediate care at the emergency department of the hospital was crucial for prognosis [32] even though at the casualty unit care, neurosurgical care for those with head trauma may be delayed [35]. Cost and limited resources in the hospital neurosurgery intensive care unit might delay surgery [32, 35]. It was reported 12% of RTI were grievous [30] while severe injury among RTC victims was reported at 36.4% [31]. For 85 crashes, 80% resulted in driver injury, and 65.9% required health care, 15.3% required in-patients with a median hospitalization time of 3 days [17]. The various forms of injuries included the Lower limbs 54%, upper limb 23%, head/face 13%, and trunk 9.8% [2]. Factors associated with mortality were; using police vehicles to hospital (*P* = 0.000), receiving medical attention within 2 to 10 h after injury (P = 0.000), 18–45 years age group (*P* = 0.019), not using helmet (*P* = 0.007), severe injuries (P = 0.000) and sustaining multiple injury (P = 0.000) [37].

### Measures to reduce the incidence of RTC

Several measures were identified to check the spate of RTC among riders. These measures include strategies to minimize crash-related injuries by improving rider safety, infrastructure and implementing effective road traffic policies [25, 28, 38]. Government investment in the improved road network, strict implementation of current road traffic regulations, and penalties awarded against anyone riding under the influence of alcohol, use of helmets, and other PPE must be made compulsory [2, 16, 28, 40] . Rider licenses should not be issued to persons who are less than the age of 25 years [32]. Random drink-driving checks for motorists by traffic police should be extended to commercial riders [26, 28, 32]. Screening for alcohol disorders among riders before they are hired or given permits as funds are made available to create road safety awareness [2, 32]. Ensuring a well-developed public transport system to help reduce RTC and youth-specific road safety programs and introducing road safety behaviors in primary school educational curriculum [16]. Road traffic police should engage riders through associations to embrace and continuously apply best road safety practices through comprehensive periodic training and retraining [15, 31, 40]. Educational intervention strategies are needed immediately to reduce associated injuries [14, 31].

## Discussion

This study described the factors associated with motorcycle-related RTC by synthesizing literature from 2016 to 2022 and highlighted the human, personal, system, regulatory elements that are associated with the higher incidence in Africa. Riders’ behavioral factors such as alcohol, drugs use, and smoking influenced the occurrence of RTC. During drug and alcohol intoxication, the sense of judgment and riders’ reaction time is reduced and increases the risk of a crash or reduces the ability of the rider to avoid oncoming danger. Human factors, including socio-demographic, physiological, and behavioral characteristics, are considered significant in influencing RTC [12, 41, 42]. The mental and physical stability of riders needs to be at an optimum function to minimize the risk associated with reduced functioning. Physio-physical stability in humans may be adversely affected by intoxication, ingestion of substances, or fatigue (physical and mental). Other studies also showed a relationship of gender and age (age between 20 and 29 years) to the chance of increased crashes among riders [41, 42]. The rider’s age had a significant relationship with alcohol and other drug use before riding in a study in Ghana [40]. The male gender and morning time riding was positively associated with RTC [43]. In Africa, people who commonly engage in commercial motor riding are mainly young males. Disability-related to this group of people can have dire consequences on economic growth and socio-demographic dynamics. Authorities must institute a concerted, coordinated training program that explicitly targets young male riders, especially those in commercial service, to avert the dangers associated with RTCs.

Furthermore, inexperience or less than 3 years of riding experience, long riding hours, excessive speeding, working late, and careless riding were associated with RTC. This on-road violation of safety precautions may result from poor regulation, carelessness, insufficient education, or increased youthful energy. Commercial motor riders’ associations in most African cities can collaborate with police and road safety authorities to regulate and ensure positive behaviors on the roads, especially by members by enforcing rules, providing proper education, efficient licensing regime, and regulated speed and time intervals for journeys. The human factors that influence RTC include violation of TRs, alcohol intake, overspeeding, wrong crossing, rash driving, playing on roads, carelessness, fatigue, or sleepiness [6, 8, 9, 12]. It is imperative that riders are given appropriate training and constantly encouraged to observe RT regulations to minimize associated risk. These are particularly essential as other studies identify lack of skill or inexperience, long riding hours, and risk-taking behaviors such as the driving speed of above 60 km/hour to be associated with the collisions of young drivers [43, 44]. The risk of severe injury related to RTC may increase with acute tiredness, sleepiness, chronic fatigue, psychoactive substance use, and careless driver behavior [6, 9, 12]. This highlights the very essence of incorporating training and retraining and ensuring continuous training of riders on road regulations and observing appropriate manners in using the roads.

This study highlighted poor knowledge, improper use of PPEs such as helmets, lack of training on TR related to the incidence of RTC. Similarly, several studies reported inadequate use of safety gadgets by commercial motor riders [6, 8, 43, 45–47]. The use of safety gadgets by riders is imperative in ensuring that riders and other road users are sufficiently protected. During riding, PPE, including helmets, protects the rider from harsh weather conditions, promotes vision, and reduces the impact of RTC [7, 10, 11]. It is also essential that riders wear the appropriate helmets to reduce severe injury that may result from RTC [11]. Pillow riders who were not using helmets had severe head injuries during an RTC, emphasizing the need for both to use the same. In some African countries like Ghana, riders must wear protective clothing like a vest, reflector, helmet, etc., when riding [40]. Authorities responsible for road safety must identify those who do not adhere to these safety precautions and appropriately sanction offenders. It is also essential to heighten education on the regulations that govern road use. The importance of rider’s license and permit with the awareness of road code, riding experience, and traffic signs were essential predictors of RTC [6, 8, 43, 45, 46, 48, 49]. In contrast, in Delta State, Nigeria, there was a non-significant association of young riders’ behavior, knowledge of road TRs, and safety measures with their involvement in RTC [49]. This emphasizes that there must be complementary efforts that ensure increased knowledge of riders and sanctioning of defaulters of TR.

Motor and road-related factors such as faulty motorcycles, bad roads, and busy intersections were associated with RTC. These demonstrate the gamut of factors that are responsible for the high rate of RTC in Africa. Vehicular mechanical faults, poor maintenance (including tires, brakes, and lights), driving old vehicles, nature of the road, time of the day, and poor weather situations were directly associated with the risk of RTC [8, 12, 45]. Riders must use aids, including those that improve vision, like the wearing of eyeglasses and body reflectors to minimize RTC. Also, road conditions (construction, surface, wet or dry), obstacles (e.g., debris on the road), and the landscape near the road was reportedly associated with RTC [12]. It was identified that the expressway, primary and secondary roads, curve road sections, roads with non-permissible marking, smooth, rut and corrugation of road surface, wee hours riding increased the probability of motorcycle and vehicle fatal crashes [50].

Some external factors like urban settings, daytime, and availability of resources influence the care that is received by victims of RTC. In low-resource countries, motor riders are more vulnerable due to the lack of protection at the scene of a crash, and as a result, victims suffer severe injuries [43, 50]. The prevalence of RTCs is between 40 and 60% among commercial motor riders in SSA, with the majority sustaining injuries [6, 12]. The lower limb was reported as a common site of fracture and injuries followed by upper limbs and skull [2, 6, 43, 45]. The strategies to be adopted to reduce the prevalence of RTC include effective policies, random drink-drug screening, increasing safety awareness, controls on issuance of permits, and enforcement of regulations [6, 43]. The TR should focus on; enforcement of speed limits to below 60 km/hour, legislation against alcohol consumption among motor riders, strict enforcement, acquisition and use of PPE, possession of valid rider’s license, and motorcycle registration [6, 44, 45].

This study harnessed the various factors associated with RTC among riders in Africa, where the trade is recently gaining popularity with its unintended consequences. The study further identified the individual rider factors related to RTC, the road and system-related factors, and the nature of treatment rendered to victims. This script is not without some limitations, as studies were limited to only English-based articles. Nonetheless, the findings can be generalized to all other parts of the continent equally exposed to cultural and economic circumstances. Also, studies that were conducted within the last 6 years were included in this study showing the time limitation as motorcycle-related crashes have remained a problem for well over a decade.

## Conclusion

This review identified various individuals, systems (road and police-related), and policy-related factors that influence RTC among riders. This study further demonstrated that RTC in Africa is multi-sectoral and multidimensional, and to control this, commensurate interventions are required. Multi-sectoral measures are imperative as interventions that minimize the spate of RTC are expected to be tailored towards commercial riders. Research must focus on identifying appropriate means to improve awareness of the protective benefits and ensure enforcement of PPEs and specifically crash helmets. The role of government and related institutions, including enforcement authorities (police), regulatory bodies, and road safety authorities, must be delineated and implemented to ensure safe road use. Concerted, coordinated, and complementary efforts from various stakeholders will improve safety on the roads as commercial cycling is increasingly gaining popularity. Intervention studies must identify appropriate means to improve road safety compliance, infrastructure, and a positive attitude towards road use. Some behavioral measures like strict regulation in issuing a license, control of alcohol use among riders, and the wearing of PPEs are imperative while increasing TR awareness is beneficial. It is also essential that research dovetail in identifying specific interventions that will help control the rate of RTC.

## Data Availability

All datasets analyzed during the current study are available from the corresponding author on reasonable request.

## Abbreviations

CINAHL: Cumulative Index to Nursing and Allied Health Literature
MeSH: Medical Subject Headings
PICO: Population, intervention, comparison/control, and outcomes
PPE: Personal protective equipment
PRISMA: Preferred Reporting Items for Systematic Reviews and Meta-Analyses
RTA: Road Traffic Accidents
RTC: Road Traffic Crash
RTI: Road Traffic Injury
SSA: Sub Saharan Africa
